# Running Neuroimaging Applications on Amazon Web Services: How, When, and at What Cost?

**DOI:** 10.3389/fninf.2017.00063

**Published:** 2017-11-03

**Authors:** Tara M. Madhyastha, Natalie Koh, Trevor K. M. Day, Moises Hernández-Fernández, Austin Kelley, Daniel J. Peterson, Sabreena Rajan, Karl A. Woelfer, Jonathan Wolf, Thomas J. Grabowski

**Affiliations:** ^1^Department of Radiology, University of Washington, Seattle, WA, United States; ^2^Centre for Functional Magnetic Resonance Imaging of the Brain, University of Oxford, Oxford, United Kingdom; ^3^Department of Neurology, University of Washington, Seattle, WA, United States

**Keywords:** cloud computing, neuroimaging pipelines, workflow, reproducibility

## Abstract

The contribution of this paper is to identify and describe current best practices for using Amazon Web Services (AWS) to execute neuroimaging workflows “in the cloud.” Neuroimaging offers a vast set of techniques by which to interrogate the structure and function of the living brain. However, many of the scientists for whom neuroimaging is an extremely important tool have limited training in parallel computation. At the same time, the field is experiencing a surge in computational demands, driven by a combination of data-sharing efforts, improvements in scanner technology that allow acquisition of images with higher image resolution, and by the desire to use statistical techniques that stress processing requirements. Most neuroimaging workflows can be executed as independent parallel jobs and are therefore excellent candidates for running on AWS, but the overhead of learning to do so and determining whether it is worth the cost can be prohibitive. In this paper we describe how to identify neuroimaging workloads that are appropriate for running on AWS, how to benchmark execution time, and how to estimate cost of running on AWS. By benchmarking common neuroimaging applications, we show that cloud computing can be a viable alternative to on-premises hardware. We present guidelines that neuroimaging labs can use to provide a cluster-on-demand type of service that should be familiar to users, and scripts to estimate cost and create such a cluster.

## Introduction

Cloud computing provides on-demand, scalable access to resources that manage, process and store data through the use of remote storage services and emulated computing systems, otherwise known as virtual machines, over the Internet. In many cases, cloud services can be used to provide access to storage and computing resources to accommodate bursts of activity without the costs of setting up and maintaining a computing infrastructure that might sit idle for a significant portion of time. For this reason, cloud computing has been widely embraced by several biomedical fields, such as comparative genomics and proteomics, and is likely to see an increase in use in these and other disciplines over the next few decades (Fusaro et al., 2011; Liu et al., 2014; Tsaftaris, 2014).

In neuroimaging, processing of datasets typically requires execution of complicated pipelines that are both time-consuming and computationally intensive. It is not uncommon for jobs to run for days, although the exact time varies depending on the type and size of the data sets being processed. Processed neuroimaging datasets can also quickly balloon in size. Most labs therefore find it necessary to purchase computer systems with enough computing power and storage space to accommodate multiple hundred-gigabyte projects and backups. However, the total cost of ownership of computing infrastructure increases non-linearly with processing requirements and the total size of data. The cost to move from a desktop to a shared-memory workstation is much smaller per added unit of processing capability or storage than the cost to move from a workstation to a large cluster. These costs motivate the examination of cloud services, which may be more suited to accommodating temporary computational bursts, to process and store neuroimaging data.

Today, popular cloud providers include Amazon Web Services (AWS), Google’s Cloud Platform, Microsoft’s Windows Azure, Rackspace’s Open Cloud and IBM’s SmartCloud Enterprise. In this paper, we limited our benchmarking to services provided by Amazon because of their development of cfncluster,^1^ an AWS framework that simplifies the creation and management of high performance computing clusters, which are similar to platforms commonly used to run large scale neuroimaging applications. Amazon offers hundreds of services, but Elastic Compute Cloud (EC2) and Simple Storage Service (S3) are among the most important in our context. While the scope of this paper is limited to AWS, we believe that the benchmarking practices described here are relevant to other cloud service providers as well.

In practice, there are a number of barriers that may deter scientists from using cloud computing services. As with any new set of complex services, there is a steep learning curve involved in learning how to use these services correctly and efficiently. Even creating an account and using the Web interface to launch EC2 instances, for example, may not be immediately intuitive. In addition, interacting with computers using a command-line interface (CLI) can be daunting. There is also overhead in terms of time and cost to package up data, automate an analysis in the cloud, and download results to a local machine. Determining which EC2 instance type and which pricing model to use can be difficult when it is not clear how these variables will affect the final cost of running a job. Moreover, many scientists may lack the interest or necessary information to make these decisions or to configure a cluster that is optimal for their workloads.

These barriers motivate our approach to define a set of best practices for deciding when it is worth running an application in the cloud. We estimate the cost of running commonly used neuroimaging pipelines on different machines and streamline parallel execution in the cloud. Specific questions we address in this paper are:

(1) Is it better to build a cluster out of larger or smaller instances, or does it not matter?
(2) Is performance across data sets stable enough such that we can use a sample of data to estimate execution time on EC2?
(3) When is it worth purchasing GPU instances for GPU-accelerated workloads rather than just using more cores?

Finally, informed by this exercise, we describe a set of tools that we have developed and made available on GitHub^2^ to help scientists estimate the cost of a job and start an on-demand customized cluster on EC2.

The benchmarking for this paper was performed in the context of a summer course held at the Integrated Brain Imaging Center (IBIC) and was made possible by a grant of credits from AWS Cloud Credits for Research.

## How: Instances and Storage

AWS EC2 provides virtual machines that are optimized for running various applications on the cloud. Virtual machines emulate computer systems, so that many virtual machines can be run on the same physical computer hardware. These virtual machines differ in the number of virtual central processing units (vCPUs) available, the size and type of memory storage used, and networking capacity. Each potential virtual machine configuration is called an “instance type.” Once an instance type has been selected and launched, it is called simply an “instance.” Up to date descriptions of available types are on EC2’s website^3^.

Pricing of EC2 instances, regardless of type, is complex. The two types of pricing that we considered here are on-demand instances and Spot instances. On-demand instances are priced per hour of usage, according to a fixed fee schedule that varies depending on the instance type and region chosen. We used on-demand pricing in our benchmarking as a worst-case scenario. Spot instances, on the other hand, allow users to bid on extra EC2 computing capacity, and is normally significantly cheaper than on-demand pricing. With Spot instances, the price fluctuates according to demand, and jobs are terminated when the cost of running them exceeds the amount that a user is willing to pay. A reasonable strategy for selecting a suitable Spot bid is to offer the on-demand rate (or slightly higher). The user will pay the current Spot rate, which is normally much lower than the on-demand rate. As long as the capacity being used represents a small proportion of the Spot market (and we would expect most neuroimaging applications to be small workloads relative to commercial interests) the bid will be unlikely to significantly affect the current Spot rate. As demand increases or decreases more broadly, the user will continue to pay the Spot rate. If the user is outbid, their instances are terminated, which is why it is safest to bid slightly more than the on-demand rate to ensure that the probability of being outbid is low.

Storage and data transfer also incur a cost. We used two types of storage in our benchmarking. The first is Amazon Elastic Block Store (EBS), which provides persistent block storage volumes for EC2 instances. This type of storage is used to store the hard drive contents of EC2 instances, along with snapshots of these instances. The second type of storage that we used is Amazon S3. Compared to EBS, S3 storage is less expensive, scalable, and offers cheap solutions for long-lived but less frequently accessed data. Although downloading data from S3 incurs a charge, transferring data into S3 is free. Moreover, accessing data stored in S3 takes less time than would be required if one were to use secure copy (SCP) to copy data from remote machines. S3 can therefore be useful for storing neuroimaging data used to run benchmarks. We did not include storage and download costs in our cost estimations because these were negligible in the applications that we evaluated. However, these are likely to vary significantly in production environments.

## When: Deciding when to Use Cloud Computing

Most neuroimaging applications are parallelizable, because a single brain, a single voxel or even sub-voxel components can often be analyzed independently of the others. Therefore, if one has multiple processing units (or cores) available, these independent analyses, or “jobs,” can be run simultaneously. In this way, the total time to execute N jobs can thus be reduced by a maximum factor of close to P, the number of processors. The original execution time of a workload divided by the reduced execution time is called the “speedup.”

Two common approaches to parallelization in neuroimaging include using a computing cluster or Graphics Processing Unit (GPU) acceleration. Computing clusters unite multiple machines with multiple cores with some scalable storage and a software platform that provides a unified way to execute parallel jobs. GPUs are special purpose processing units that are optimized for processing data in parallel, and are generally better at handling certain types of parallel compute-intensive processing than general purpose Central Processing Units (CPUs). Many long-running neuroimaging applications have GPU-enabled versions to accelerate processing. Examples include Oxford Centre for Functional MRI of the Brain (FMRIB) Software Library’s (FSL) BEDPOSTX and PROBTRACKX for tractography (Behrens et al., 2003b, 2007; Jbabdi et al., 2012), and FreeSurfer, a workflow to quantify structural measures such as cortical thickness (Fischl, 2012).

We assume that because neuroimaging is a compute-intensive field, most neuroimaging scientists have access to some on-premises (i.e., dedicated) computing platform. Having access to even more processors through the cloud means that either the time to execute a particular analysis can be reduced, or that a larger problem size (e.g., more subjects) can be solved in the same amount of time. Analyses that take too long to be feasible are “intractable.” Cloud computing, by providing temporary access to specialized hardware or bursts of computational resources, is therefore attractive when it can make an analysis that is intractable tractable more cheaply than by purchasing dedicated hardware.

Several trends in neuroimaging make cloud computing an attractive option. These include:

(a) The advent of data-sharing initiatives, such as the Alzheimer’s Disease Neuroimaging Initiative (ADNI)^4^, the 1000 Functional Connectomes Project/INDI^5^, the UK Biobank^6^ (Miller et al., 2016), and the Human Connectome Project (HCP)^7^ (Van Essen and Ugurbil, 2012; Sotiropoulos et al., 2013), that have made several large data sets available to the public. Most of these data sets come from multi-site collaborative projects. Therefore, each data set may include more subjects than most individual studies could expect to obtain, and sites may not have the computational power to process these data sets, or the storage to hold both raw data and data products, providing an incentive to use cloud services. Even when data are shared under license agreements that prevent making these data publicly available, researchers may benefit from storing and processing them securely on cloud resources.
(b) Recently developed multiband echo planar imaging (EPI) approaches can greatly accelerate acquisition of magnetic resonance imaging (MRI) data, yielding images with higher spatial and temporal resolution than those achieved using standard EPI protocols (Moeller et al., 2010). However, these scans require more time to process and greater storage space.
(c) The use of non-parametric statistical methods for voxelwise and clusterwise inference are becoming increasingly popular in the neuroimaging community (Eklund et al., 2016a,b). Most of these methods involve permutation testing, a technique that shuffles data randomly and re-runs the statistical analysis on each permutation to develop a distribution of the test statistic under the null hypothesis. Permutation testing is computationally intensive, because one needs to execute hundreds or thousands of statistical tests instead of a single test to obtain a distribution. Cloud resources can potentially speed up execution if tests are run simultaneously over a cluster of virtual machines with multiple cores (Winkler et al., 2014).

Because on-premises computing resources may be scarce, there has been significant interest in using cloud computing tools for processing neuroimaging data (Mori et al., 2016; Shatil et al., 2016; Vogelstein et al., 2016). The Neuroimaging Informatics Tools and Resources Clearinghouse (NITRC), for example, is a popular repository for neuroimaging tools and data that began offering a cloud-based virtual computing platform in late 2012. The NITRC Computational Environment (NITRC-CE) comes pre-configured with many widely used neuroimaging analysis applications and an easy-to-use graphical user interface (Kennedy et al., 2015). Similarly, C-PAC (Configurable Pipeline for the Analysis of Connectomes) is an environment to automate preprocessing and analysis of resting-state fMRI data, and is available as a machine image on EC2 (Craddock et al., 2017). Newer platforms like MRICloud shift neuroimaging processing entirely to the cloud, and links different types of service tools to offer an integrated software-as-a-service model that enables users to run analyses and quality assurance procedures through a web interface (Mori et al., 2016).

### The Total Costs of Ownership of Different Computing Platforms

We considered three scenarios for parallel execution of neuroimaging applications. The first is a commodity 4 core *desktop*. We imagine that this might be the type of machine that a researcher purchases to use for interactive work, and is largely self-maintained and administered. The second type of machine is a 24 core shared memory *workstation*. This type of workstation might service a lab, and is modeled on typical workstations here at IBIC. Associated costs to maintain such a workstation include a yearly support contract, hosting in a shared rack with uninterruptable power supply, and maintenance by a system administrator (for some percentage of effort). Finally, the third machine is a scalable *cluster*, with a high performance file system. Associated costs to maintain such a cluster are based on costs for the University of Washington Hyak system, a shared and high performance computing cluster dedicated to research computing. Pricing for Hyak is highly subsidized, but includes power, networking, and system administration support. Descriptions of all machines and how we arrived at our cost estimates can be found in Supplemental Materials.

**FIGURE 1:**
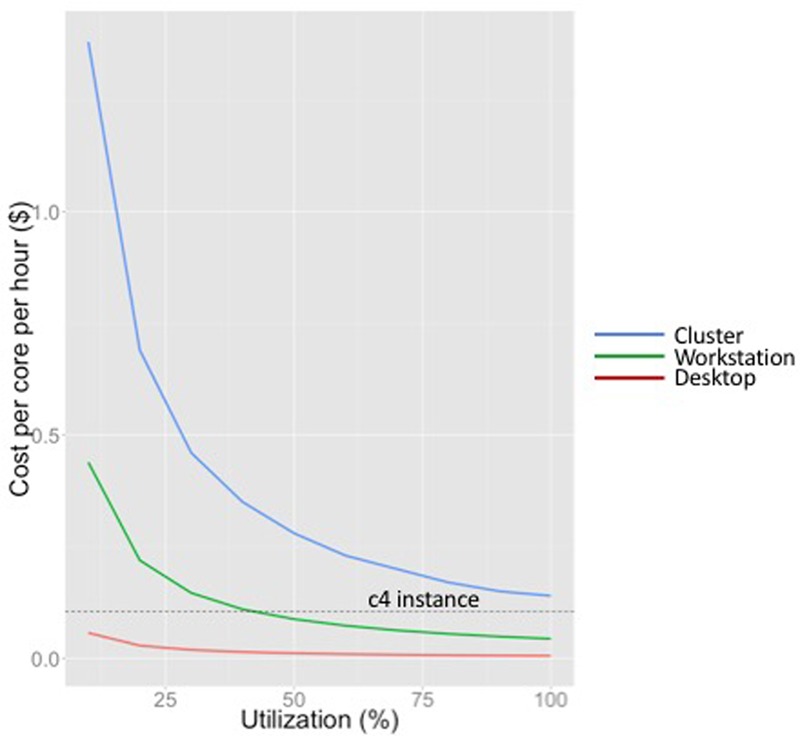
Cost per core per hour for different computing platforms at a range of utilizations (desktop, workstation and cluster). Cost of an on-demand c4 instance (not dependent on utilization) is shown by the dotted line.

**Figure 1** shows the costs per hour per core for each of these three models. We amortized the cost over 5 years for the workstation and desktop (matching a typical grant cycle), and over 4 years for the cluster (as Hyak does). As seen in **Figure 1**, the cost per core-hour increases dramatically with scale across this range of computation platforms. This is largely because of system administration overhead and system complexity. We also note that because computer time is wasted if not used, the cost per core-hour decreases for every computation platform as utilization increases.

The equivalent on-demand cost per core-hour for a compute-optimized EC2 instance (c4) in the us-west-2 region (as of this writing) is indicated by the horizontal line in **Figure 1**. Note that the compute capacity of EC2 instances is specified in terms of vCPUs, each of which is a hyper-threaded CPU. Hyper-threading duplicates certain sections of a physical core, but not the main execution resources, creating two virtual cores for each physical core. Although this improves throughput for many applications, neuroimaging workloads are too computationally intensive to benefit significantly from hyper-threading. In this plot we estimate the cost of a single real core as two times the cost of a vCPU on a c4 instance (as of this writing, this is $0.105/hour in the us-west-2).

**Figure 1** shows that the workstation we have designed for neuroimaging applications is competitive with cloud computing at approximately 40% utilization. As we describe in the next section, if a job is intractable on a typical workstation (characteristic of those found in neuroimaging labs such as IBIC), and does not reflect an ongoing increase in workload that would push utilization consistently over approximately 70%, we would consider cloud services before investing in cluster infrastructure. We do not have sufficient expertise with GPUs to draw a similar performance curve for GPUs, although one could certainly do so to evaluate the utilization at which it was worth investing in GPUs versus using GPU-enabled instances.

None of these cost estimates explicitly include uninterruptible power supply costs, cooling, networking costs, or space, as these are subsidized by the university. If included, they would not change the shape of the curves, but they would significantly increase the utilization until which cloud computing is an attractive alternative to on-premises resources. Also, all EC2 cost estimates were based on AWS’ on-demand pricing. Spot pricing for EC2 instances is much lower (although more volatile), and can change the point at which cloud computing using AWS is cost-effective. As of this writing, 30-day Spot pricing estimates for c4 instances were approximately 20% of the on-demand pricing, which makes cloud computing using AWS exceptionally attractive.

Finally, the cost estimates do not include storage (or transfer), which is charged per gigabyte per month, and varies dramatically by lab and analyses. An attractive benefit to maintaining a workstation, even if it experiences less than 40% utilization, is that it provides redundant storage for 20 TB of neuroimaging analyses. At current pricing in the us-west region, it costs $23 per month to store 1 TB of data on S3 for standard, or frequent, access (or approximately $5520 per year for 20 TB). Using one of our own current projects with anatomical, diffusion, perfusion and multiecho fMRI sequences as an example, we estimated that a project with 100 subjects would require 105 GB of storage space for processed FreeSurfer data, which costs $2.42 per month to store on S3. Unprocessed data for 100 subjects would need approximately 177 GB of storage space, costing $4.07 per month on S3. Once data has been processed, the size of the data increases dramatically to 1.54 TB. This would cost $35.37 per month on S3. Adding up costs, we would expect a typical project to cost about $38/month to store on S3 once data has been processed, not including the storage of scripts and programs that may be necessary to process the data and snapshotting of the instances on EBS. Maintaining data on EBS or S3 storage over time can thus dramatically change the cost estimates of **Figure 1**.

### The Time Required to Analyze Data on Different Platforms

**FIGURE 2:**
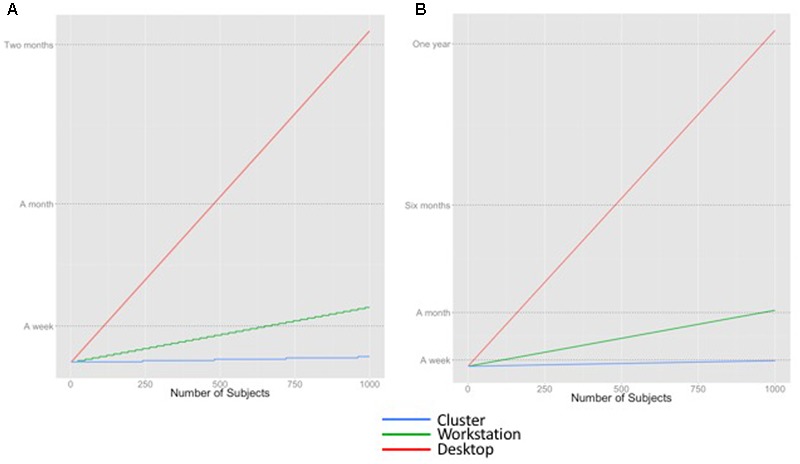
Estimated time to execute different workloads on different computing platforms. **(A)** FreeSurfer **(B)** Whole brain tractography of HCP data.

**Figure 2** shows the theoretical execution time curves for two applications: (1) FreeSurfer, a pipeline used for cortical parcellation, and (2) whole brain tractography on HCP data, on our identified compute platforms. FreeSurfer takes approximately 6 h to process one brain on a single workstation, assuming no within-brain parallelism, i.e., the total execution time across multiple cores is limited by the processing time of a single brain. Whole brain tractography takes approximately 31 h to process one brain, but the processing can be fully parallelized, yielding smooth curves.

**Figure 2** illustrates that the time required to run an analysis application, the number of subjects in the data set(s) being processed, and the available computing resources all contribute to the estimate of execution time. For example, a lab with a modest cluster of 240 cores (e.g., 10 workstations) will be able to process 1000 subjects with FreeSurfer in a week. In contrast, that same workload would monopolize a desktop for months. Whole brain tractography, a procedure that takes approximately five times as long as FreeSurfer’s recon-all to run to completion, would take a week to complete on a cluster, and would probably be considered intractable on a workstation.

There are GPU-enabled versions of FSL’s BEDPOSTX and PROBTRACKX, FSL utilities used in our tractography script, which offer speedup that may be larger than the number of cores available on a node. FreeSurfer also released a GPU-enabled pipeline in 2012, but this is not actively supported anymore; consequently, we did not benchmark FreeSurfer on EC2 GPU-enabled instances. GPU acceleration is not reflected in the calculations in **Figure 2**; we discuss the tradeoffs between using a GPU instead of a CPU more extensively in the results.

In **Figure 2**, we assume perfect speedup, i.e., that the time to execute N jobs on P cores is exactly N/P. In reality, this is not true. Even though the N jobs are independent, as P increases there may be aspects of the system architecture that serialize part of the computation and limit performance. More importantly, there is additional human time that is required to set up and check the results of a large parallel analysis. There is, unfortunately, no way to purchase computer resources to reduce this effort. You might decide that it does not matter whether an analysis finishes in an hour or a week if you will not have the time to look at the results for 2 weeks. However, running hundreds or thousands of tractography analyses on your desktop is likely to take too long to be feasible simply because P is too small.

In summary, the decision of when to run an analysis in the cloud depends upon (1) the number of subjects, (2) the time it takes a program to run for each subject on-premises, (3) how the workload can be parallelized (potentially using GPUs), and (4) the human overhead involved in setting up and checking the analysis, which cannot be parallelized.

## At What Cost: Benchmarking EC2

Many variables contribute to the performance of an application on EC2 instances, and benchmarking cloud services is an important research topic in its own right (Deelman et al., 2008; Leitner and Cito, 2014). While there are published methods with detailed steps for evaluating cloud services, variation in performance of cloud services over time can make it difficult to generalize results from a paper (Gillam et al., 2013). Performance may vary because of characteristics that are transparent, such as the match of basic application characteristics to instance type, the time of execution, and geographic region. Performance may also vary because of characteristics that are not clearly visible to the user, such as the hardware heterogeneity that underlies virtual instances, scheduling policies used by the cloud provider, and the effects of running multiple virtual instances together with other users on the same hardware (otherwise known as multitenancy). Finally, performance can be unpredictable because of the characteristics of the application itself (Minervini et al., 2015). The variability of execution time and its impact on cost must also be considered.

Given our limited benchmarking budget, we focused exclusively on benchmarking our application suite on appropriate instance types. We excluded certain instance types from consideration because they were mismatched to our sustained CPU-intensive workloads. For example, the t2 instance types are intended to handle short bursts of CPU utilization rather than sustained CPU intensive workloads. To achieve high overall CPU utilization, a single physical core would have to support multiple t2 instances which are then penalized by the virtual machine scheduler for sustained CPU usage. For this reason, we did not examine t2 instance performance. We also did not look at instance types intended for memory-intensive or storage-intensive applications. Instead, we limited our evaluation of instance types to the latest generation of general purpose and CPU-optimized instance types as of this writing (m4 and c4). Besides this, we evaluated GPU performance on g2 instances (the latest AWS GPU instance type as of this writing, which was an NVIDIA GRID K520). A complete list of EC2 instance types with detailed descriptions and use case scenarios can be found on Amazon’s EC2 website^8^.

To estimate the costs of running fairly standard neuroimaging applications on different instances, we benchmarked execution time on each instance type (using all available vCPUs in parallel) in two conditions. In the first condition, we processed the same data set (i.e., same brain) on each vCPU, using all vCPUs in the instance. In the second condition, we processed a different data set on each vCPU, using all vCPUs in the instance. This allows us to examine how variable execution time is for different data sets. In each condition, we calculated the average time for the processing of the data sets to complete on the fully loaded instance. This allowed us to determine whether it was reasonable to use an execution time estimate from a single data set (or an average) as a proxy for multiple data sets. Note that AWS charges for instances by the hour; in our cost estimates we did not “round up” the execution time for jobs that took less than an hour. This overhead would be small under heavy utilization.

We did not conduct our benchmarking on cfncluster, although the appeal of using cfncluster and Spot pricing motivated this work. As in our normal workflow, we prototype and benchmark on single machine and run on a cluster in production. There is some fixed setup time for cfncluster creation that may ultimately need to be considered in total cost when running on a cluster.

Given that there are many other sources of performance variability that we did not control or evaluate, our benchmarking is admittedly not comprehensive. It is intended instead to represent a strategy for obtaining information to guide a realistic and generalizable cost analysis using critical neuroimaging applications at a specific lab.

### Description of Benchmarks

Assuming that different labs have different classes of hardware to conduct the analyses that they do, and that most scientists might prefer to conduct analyses that complete within a few days, we identified three candidate benchmarks for AWS. These are described below.

*FreeSurfer* is an image analysis suite designed for processing structural neuroimaging data, which performs cortical reconstruction and volumetric segmentation (Fischl, 2012). The technical details of these procedures are described in prior publications (Dale and Sereno, 1993; Dale et al., 1999; Fischl et al., 1999a,b, 2001, 2002, 2004a,b; Fischl and Dale, 2000; Ségonne et al., 2004; Han et al., 2006; Jovicich et al., 2006; Reuter et al., 2010, 2012). Briefly, this processing includes motion correction and averaging of T1 weighted images (Reuter et al., 2010), removal of non-brain tissue using a hybrid watershed/surface deformation procedure (Ségonne et al., 2004), automated Talairach transformation, segmentation of the subcortical white matter and deep gray matter volumetric structures (Fischl et al., 2002, 2004a), intensity normalization (Sled et al., 1998), tessellation of the gray matter white matter boundary, automated topology correction (Fischl et al., 2001; Ségonne et al., 2007), and surface deformation following intensity gradients to optimally place the gray/white and gray/cerebrospinal fluid borders at the location where the greatest shift in intensity defines the transition to the other tissue class (Dale and Sereno, 1993; Dale et al., 1999; Fischl and Dale, 2000). We used FreeSurfer version 5.3 to conduct this benchmarking.

FreeSurfer typically takes several hours or more to process a single brain, which means that an analysis of several hundred subjects will be faster if subjects are run in parallel on a large cluster.

Two data sets were used for benchmarking FreeSurfer. One set of isomorphic 1mm structural images was obtained from subjects in the Alzheimer Disease Neuroimaging Initiative (ADNI-1 & ADNI-Go^9^). The ADNI was launched in 2003 as a public-private partnership, led by Principal Investigator Michael W. Weiner, MD. The primary goal of ADNI has been to test whether serial magnetic resonance imaging (MRI), positron emission tomography (PET), other biological markers, and clinical and neuropsychological assessment can be combined to measure the progression of mild cognitive impairment (MCI) and early Alzheimer’s disease (AD). For up-to-date information, see www.adni-info.org. This data set was processed using the standard recon-all pipeline on a single structural scan.

The second data set consists of.8mm isomorphic structural images scans from the University of Washington’s Alzheimer’s Disease Research Center^10^ (ADRC). The ADRC dataset was run through FreeSurfer twice; once on downsampled data (resolution = 1 mm), and the second time on the original high-resolution 0.8 mm data, using the procedure described by Lüsebrink et al. (2013).

*Tractography* is a technique used to process diffusion MRI data to quantify the structural connections between regions in the brain (Mori et al., 1999). Specifically, we benchmarked probabilistic tractography on data from the Human Connectome Project (HCP: Van Essen et al., 2012). To do this, we used FSL’s BEDPOSTX (Bayesian Estimation of Diffusion Parameters Obtained using Sampling Techniques) to build up distributions of diffusion parameters obtained at each voxel (Behrens et al., 2003b, 2007) using FSL’s pre-specified default parameters, then ran the outputs through FSL’s PROBTRACKX (Behrens et al., 2003b, 2007). PROBTRACKX generates probabilistic streamlines by repeatedly sampling data from the distributions of the principal diffusion directions obtained from BEDPOSTX. As an example of a realistic application of probabilistic tracking, we used PROBTRACKX to compute a frontal lobe connectivity-based parcellation of the thalamus, which involved classifying each voxel in the left thalamus according to the strength of the connection from the voxel to left frontal lobe regions in the Desikan-Killiany atlas (Behrens et al., 2003a; Desikan et al., 2006) (PROBTRACKX thalamic parcellation). For comparison with published results, we also timed the application reported by Hernandez-Fernandez et al. (2016), which reconstructs dense connectome matrices (PROBTRACKX dense connectome). Both benchmarks used data from the WU-Minn HCP 500 Subjects release.

**FIGURE 3:**
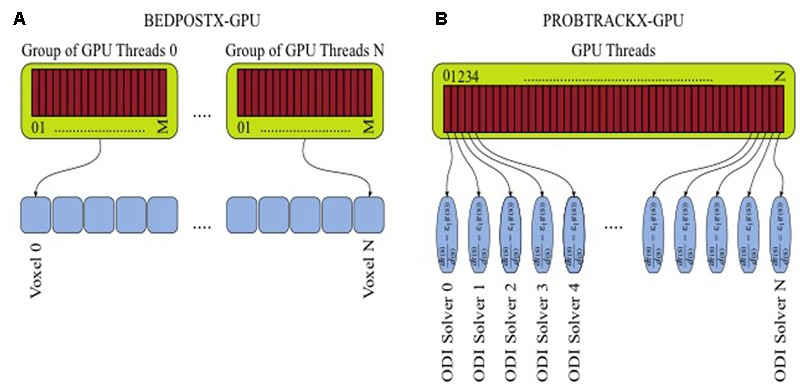
Parallelization and multithreading for tractography. **(A)** GPU-enabled BEDPOSTX. **(B)** GPU-enabled PROBTRACKX.

These methods are computationally very demanding. BEDPOSTX uses a computational modeling and signal-processing framework, and takes as its input multidimensional data that can consist of millions of image voxels. A Bayesian inference framework and inversion of models is then performed via Markov-Chain-Monte-Carlo (MCMC) integration. Such a problem is very parallelizable given the large number of independent elements that can be subjected to voxel-wise modeling. In the GPU version, a further parallelization occurs during the estimation of the a-posteriori distributions of model parameters at each voxel, which involves expensive likelihood calculations. Thus, several lightweight threads collaborate together to execute within-voxel computations (**Figure 3A**).

In PROBTRACKX, another inference framework is used to numerically integrate the local estimates from BEDPOSTX for the purposes of solving ordinary differential equations (ODE). The task is repeated many times in a Monte-Carlo fashion. Millions of ODE solvers need to be launched in order to achieve converged spatial distributions, and this can lead to large computational times. However, solvers can be computed in parallel and be assigned to different threads (**Figure 3B**). Indeed, the GPU implementations of BEDPOSTX and PROBTRACKX can allow speedups of more than two orders of magnitude compared to the single-threaded CPU implementations. To benchmark tractography, we used a GPU-enabled version of BEDPOSTX included in FSL version 5.0.9 (Hernández et al., 2013) and a beta version of GPU-enabled PROBTRACKX (Hernandez-Fernandez et al., 2016).

The third application we benchmarked was *Neuropointillist*, an in-house program written in R (version 3.2.3) to analyze neuroimaging data at the group-level using mixed effects modeling. The Neuropointillist R package defines functions to combine multiple sets of neuroimaging data, automate parallel execution of arbitrary R code (a “model”) on each voxel, output results, and reassemble the data. Although still undergoing active development, we decided to benchmark Neuropointillist because it is an example of a compute-intensive voxel-wise analysis that is analogous to voxelwise analyses in other labs.

Neuropointillist was benchmarked on EC2 using a dataset obtained from John Flournoy (personal communication; Pfeifer et al., 2013). Adolescents were scanned at 3 waves (N1 = 78, N2 = 49, N3 = 35) at ages 10–16 while making evaluations of target ‘self’ and ‘other’ (Harry Potter) in both social and academic domains. An equal number of items that had positive and negative valence were presented to participants during each MRI scan session, and sample phrases included: “I am popular,” “I wish I had more friends,” “I like to read just for fun,” and “Writing is so boring.” The data set consisted of whole-brain *t*-statistic maps for each cell of a 2 × 2 Analysis of Variance (ANOVA) design with target (‘self’ or ‘other’) and domain (social or academic) as factors, as well as demographic information such as age, sex, pubertal status and answers from self-report questionnaires. We fitted and compared two mixed effects models across a sample of voxels in the brain; the base model included fixed effects of age, time, domain and target and a random intercept for each individual and time point, while the extended model included an additional interaction between target and domain. Equations for these models are included in Supplemental Materials. The two models were compared using the ANOVA function in R to determine where in the brain an interaction between target and domain was present. Timings for Neuropointillist are for a “job,” or a subsample of voxels, not an entire brain.

**Table 1** shows the full complement of benchmarks and instance types that we benchmarked.

**Table 1:** Benchmarking matrix.

Application	Data type	EC2 instance type							
benchmarked		m4.large	m4.xlarge	m4.4xlarge	c4.large	c4.xlarge	c4.4xlarge	c4.8xlarge	g2.2xlarge
Freesurfer Recon-all	Same	X		X		X		X	
(Downsampled Images)	Different	X		X		X		X	
Freesurfer Recon-all	Same	X		X		X		X	
(High Resolution Images)	Different	X		X		X		X	
FSL PROBTRACKX	Same								X
	Different								
FSL BEDPOSTX	Same								X
	Different								X
Neuropointillist	Same	X	X	X	X	X	X		
	Different	X	X	X	X	x	X		

## Results

### Choosing Instance Types to Obtain the Best Performance

**FIGURE 4:**
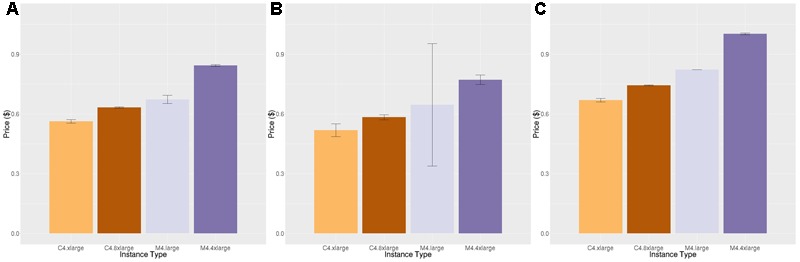
Price per brain ($) to execute FreeSurfer. **(A)** Price per brain for identical subjects. **(B)** Price per brain for different subjects. **(C)** Price per brain on identical subjects using only half of the available cores. Vertical bars show 95% confidence intervals, calculated over the number of jobs equal to the number of vCPUs **(A,B)** or half the number of vCPUs **(C)**.

**FIGURE 5:**
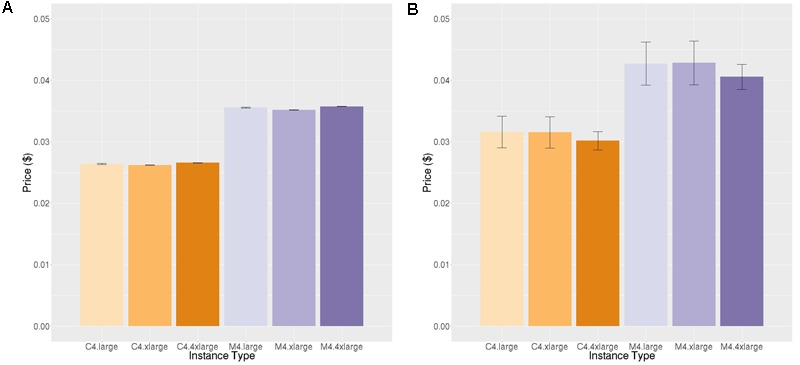
Price per job ($) to execute Neuropointillist. **(A)** Price per job for same workload **(B)** Price per job for different workload. Vertical bars show 95% confidence intervals, calculated over a number of jobs equal to the number of vCPUs.

**Figures 4, 5** show the price per brain to execute FreeSurfer and Neuropointillist, two examples of relatively CPU-intensive applications, on a range of m4 and c4 instances. We see in both cases that the c4 instances are less expensive per brain than the m4 instances. The c4 instances have less memory and cost less per vCPU than the more general-purpose m4 instances. Hence, if applications can run with limited memory requirements, they can execute more cheaply on c4 instances. The processors in c4 instances are also faster than those in m4 instances, as indicated by the description of instance types.^11^ We find execution time to be slightly less on the c4 instances than on the m4 instances, contributing to the lower price per brain on c4 instances. Note that there can be substantial variability in individual subjects, as in the subjects selected for benchmarking on the m4.large instance. By chance, one subject completed significantly faster than the average. With larger samples such execution time differences were not significant.

In addition, we noted that the price per brain for FreeSurfer was systematically lower on smaller instance types within a class of instances (i.e., c-class instances or m-class instances) than on larger instance types, despite the fact that the price per vCPU is identical across instance types within a class. One possibility that we explored for this performance differential is the fact that two vCPUs correspond to a single physical core, and the vCPUs are implemented using hyper-threading. Hyper-threading is typically not helpful for CPU-intensive jobs of the type we are benchmarking. We hypothesized that on small instance types that reflect a portion of a larger physical machine, the penalty for hyper-threading might be smaller than on larger instance types that occupy the entire physical machine (except for cores reserved for the hypervisor, which creates and runs virtual machines). To test this hypothesis, we ran the FreeSurfer benchmark using only half the number of vCPUs (**Figure 4C**). Although execution time decreased across all instance types, it decreased by less than half, and the cost pattern we observed was the same.

A technical feature that would explain this performance is that AWS uses intel Xeon processers which supply Intel Turbo Boost technology. Turbo Boost increases the chip clock speed based on temperature and power consumption of the cores. This feature is enabled through the virtualization layer that maps the virtual instances to the physical server. Turbo Boost does not engage when the entire server is used for computationally heavy workloads as is the case when all of the processors are being used on a c4.8xlarge. However, when the physical server is shared among other workloads, as it is in smaller instances, it is possible that the overall server utilization is lower and Turbo Boost can engage, improving performance of our jobs on smaller instances relative to larger ones. This would mean that this effect is not reliable, because it is dependent upon load on the physical server. We did not see this performance differential across instance types on neuropointillist, but those jobs may be too short to witness this effect. We did not have the budget to test this hypothesis further.

**FIGURE 6:**
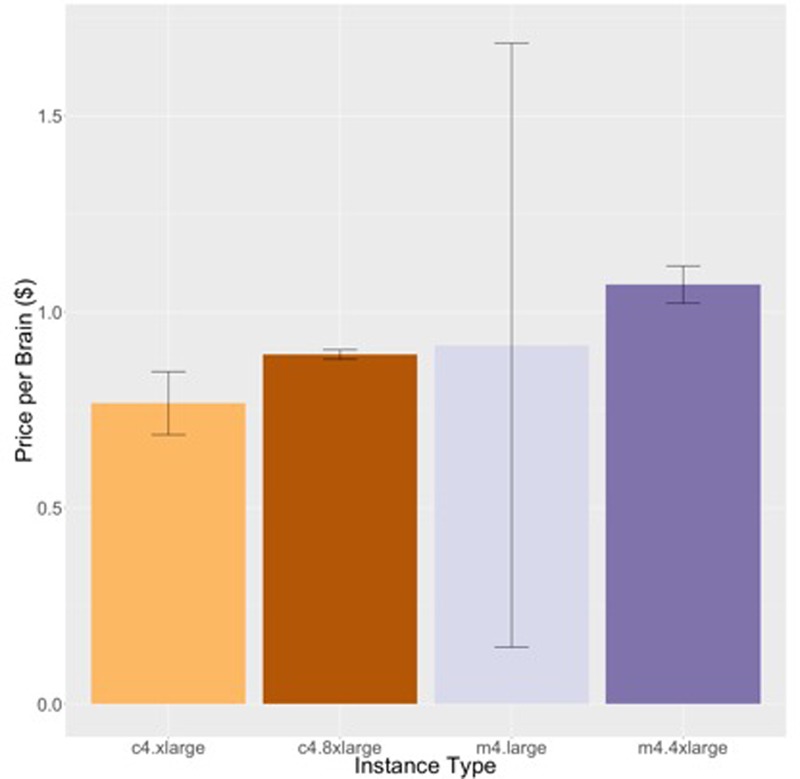
Price per brain ($) to execute the downsampled portion of the high-resolution FreeSurfer pipeline on different subjects. Vertical bars show 95% confidence intervals, calculated over a number of jobs equal to the number of vCPUs.

**FIGURE 7:**
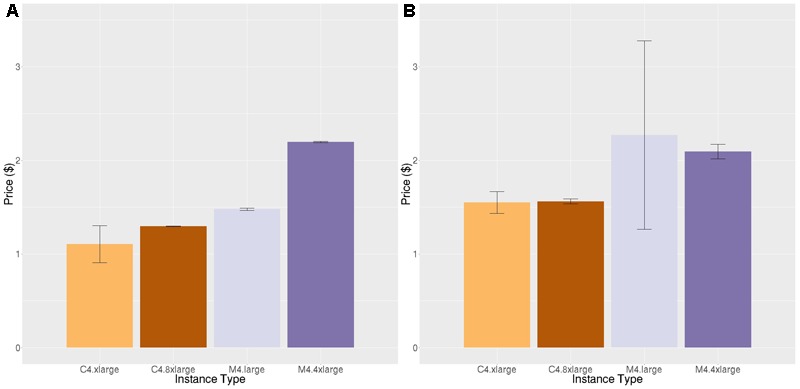
Price per brain ($) to execute the high-resolution portion of the high-resolution FreeSurfer pipeline. **(A)** Price per brain for identical subjects **(B)** Price per brain for different subjects. Vertical bars show 95% confidence intervals, calculated over a number of jobs equal to the number of vCPUs.

**Figures 6 and 7** show the results for the two stages of the FreeSurfer high resolution pipeline. The downsampled run (**Figure 6**) is analogous to the timing in **Figure 4**, but on a different data set. We can see that although overall execution time and cost is slightly higher for the higher resolution ADRC data than the ADNI data, the rank ordering of instance class costs is the same for both stages of processing. Note that we do not see as strong an effect of smaller instance types being more efficient.

### Estimating Execution Time on EC2

**Table 2** shows the ratio of execution time on our workstation to the mean execution time on all c4 and all m4 instances for the same subject. We grouped instance types to compute this ratio because performance variability across instance types was relatively small. On average, the ratio of the workstation execution time to c4 instances is higher than the ratio of workstation execution time to m4 instances, reflecting the fact that the c4 instances are slightly faster. We observe that across the two applications, the ratio of execution times is relatively stable (especially for m4 instances). The ratio of workstation time to execution time on c4 instances is somewhat more variable. This may be related to the variable impact of Turbo Boost technology as described above. However, this general stability shows that we can estimate execution time of compute-intensive applications on EC2 from on-premises resources to within a reasonable error.

**Table 2:** Ratio of execution time on reference workstation to AWS (using the average of timings on all c4 and m4 instances together), and timings on all c4 and m4 instances separately.

Application	Workstation/AWS	Workstation/c4	Workstation/m4
Neuropointillist	0.65	0.71	0.60
FreeSurfer	0.60	0.61	0.59
FreeSurfer High Resolution Pipeline, Stage 2	0.63	0.64	0.60

### GPU Acceleration versus Increased Parallelism

The applications used for tractography (BEDPOSTX, PROBTRACKX) can use GPUs, where available, to accelerate computation. However, instance types that support GPUs are typically more expensive than compute-optimized or general purpose instance types. This begs the question of when it is cheaper to use more expensive GPU-enabled instances for a shorter period of time versus a larger number of cheaper non-GPU-enabled instance types for a longer period of time, if the goal is to obtain the highest throughput at the lowest cost.

**FIGURE 8:**
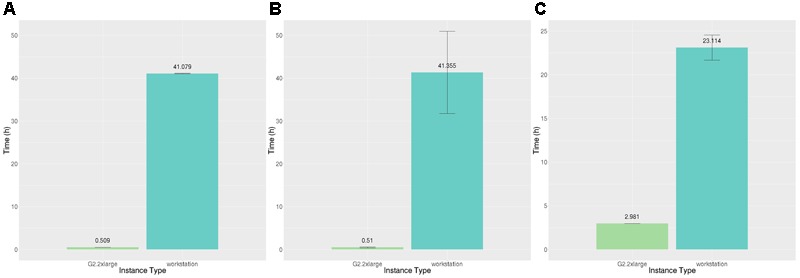
Time (hours) to execute BEDPOSTX and PROBTRACKX on our reference workstation and two AWS GPU-enabled instances. **(A)** Execution time (hours) for identical subjects. **(B)** Execution time (hours) for different subjects. **(C)** Execution time (hours) to execute PROBTRACKX on identical subjects. Vertical bars show 95% confidence intervals calculated over *N* = 8 runs.

The answer to this question depends upon the speedup achievable using GPUs versus non-GPU-enabled instances and the relative costs of these instances. If the relative speedup obtainable is not substantially higher than the relative cost, it is not worth the expense of GPU instances. We estimated the time to run BEDPOSTX and PROBTRACKX on non-GPU-enabled instances by scaling execution times on our reference workstation. **Figure 8** shows the execution time to execute both BEDPOSTX and PROBTRACKX sequentially on our reference workstation and on a g2 instance with a single GPU. BEDPOSTX runs 80.9 times faster on a GPU than on a single workstation processor, which we determined from our benchmarking (see **Table 2**) is conservatively 1.67 times the speed of a c4 vCPU. Therefore, we estimate that the speedup relative to a vCPU would be 1.67^∗^80.9, or approximately 135. **Figure 8C** shows the time to execute PROBTRACKX on our example problem on a workstation and a single GPU. PROBTRACKX runs only 7.8 times as fast on this problem on a GPU than on a single workstation processor. Thus, the speedup relative to an equivalent vCPU would be approximately 1.67^∗^7.8, or 13.0. This was substantially less than the speedup of 68.29 reported by Hernandez-Fernandez et al. (2016) so we also ran the dense connectome benchmark described therein on our workstation and on the same GPU platform.^12^ The speedup of the dense connectome benchmark (calculated from only one timing on each platform) was 22.9 (35.6 h on our workstation and 1.55 h on a single GPU). This would be equivalent to a speedup of 38.2 (1.67^∗^22.9) relative to a vCPU.

**FIGURE 9:**
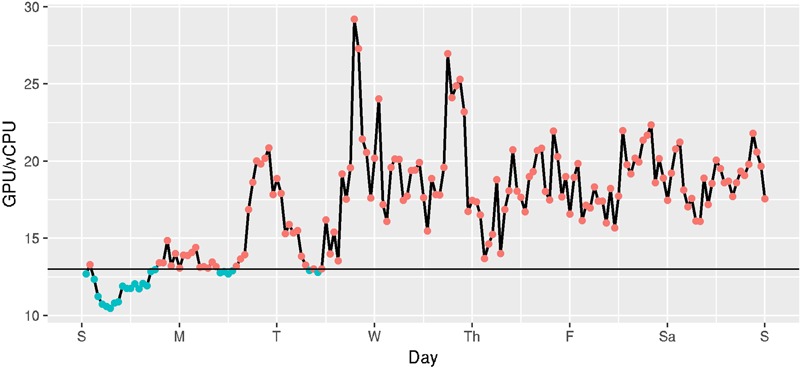
Hourly ratio of cost per GPU to cost per vCPU in US regions, estimated hourly over a week (Oct 23 2016 - Oct 29 2016). Hours below the horizontal line show when it is more cost-effective to run PROBTRACKX on a GPU versus the vCPUs available on EC2 instances.

In AWS’ Spot pricing system, the ratio between the cost of a GPU and a vCPU fluctuates as the prices for those instances changes over time. **Figure 9** shows the ratio between the minimum cost for a GPU (on a g2 instance) and the minimum cost for a vCPU (on an m4 or c4 instance) in US regions for the week of October 23 through October 29 2016. This information is important to understand whether a GPU accelerated program would be cheaper to run on GPU instance types or vCPUs. We need to look up the speedup obtainable for a GPU to a vCPU for a particular application and determine what fraction of the time the price ratio is less than the speedup.

For example, we estimated that BEDPOSTX runs approximately 135 times faster on a GPU-enabled (g2) instance than on non-GPU-enabled instances (m4, c4), on our HCP subjects. The GPU/vCPU price ratio even of on-demand instances is far less than 135, so it will always be cheaper to run BEDPOSTX on GPU-enabled instances on this workload. However, the speedup of PROBTRACKX on our benchmark was only 13. A horizontal line shows where this line occurs in **Figure 9**. Referring now to **Figure 9**, we can see that of the 168 h in the week, only 23 (13.7%) had a cost ratio favorable to the g2 instances. On average, it would be more expensive to execute this PROBTRACKX benchmark on GPU-enabled instances than on vCPUs. In contrast, the speedup of PROBTRACKX relative to a vCPU on the dense connectome benchmark was much higher, at 38.2. At this speedup, the cost ratio was favorable to the dense connectome benchmark.

## Tools to Implement Best Practices

Through the process of conducting this benchmarking, we developed some tools to help replicate this approach. These are publically available and documented at https://github.com/IBIC/ibic-cfncluster. They are summarized here.

### Estimating and Minimizing Cost

We were able to calculate the ratio of execution time of CPU-intensive programs on a single core on our workstation to an m4 or c4 vCPU on EC2. This ratio allowed us to estimate the number of vCPU hours that are required to run a job on EC2. We wrote a script (get_spot_estimate) to use the AWS interface to query the Spot pricing history for the previous week to identify the historically least expensive instance type that could be used to create a cluster and estimate the expected and maximum cost to execute the job on that cluster.

The inputs to the script are the number of jobs, and the expected number of hours on a vCPU. It is possible to produce cost estimates at a different site by running benchmarks on an on-premises reference workstation (using local storage) and on EC2 to obtain a site-specific scaling factor.

### Creating Specialized Clusters on Demand

In this paper we consider the scenario where EC2 services are used to speed execution of a problem through parallelism. Although we did our benchmarking by launching instances individually and using secure shell (ssh) to connect to them, to run a large-scale application one must create a cluster of computers. This additional configuration is automated by the AWS cfncluster package. We followed Amazon’s recommendations for best practices to use this package to automate creation of a configuration tailored for a specific application.

The recommended workflow is to create a cluster of cfncluster default Amazon machine images (AMIs), and to write a configuration script that installs only the software required for the application. Data is also most quickly transferred to and from S3. Once the cluster is created, a job can be run using one of the supported schedulers, including Son of Grid Engine (SGE; Gentzsch, 2001)^13^, torque (Staples, 2006), Openlava (Teraproc Inc.), and SLURM (Jette et al., 2002). We have used only SGE in our testing. Note, however, that AWS imposes limits on the number of instances that can be started by an individual by default. Individuals must request these limits be increased to create a large cluster.

cfncluster can take advantage of Spot pricing, using the cheapest instance type identified from the script described in Section “Choosing Instance Types to Obtain the Best Performance,” and also dynamically launch and stop instances as needed to service the queue. With Spot pricing, instances may be terminated if Spot prices rise above your bid price, when the demand for Spot instances rises, or when the supply of Spot instances decreases. Because we normally execute our parallel workloads using qmake, a parallel version of make, it is easy to pick up from partially completed jobs should instances be terminated (Askren et al., 2016). However, if the computational time for each step (before output or checkpointing information is saved) is much longer than an hour, the cost for this compute time would be lost. Similarly, if Spot instances are not available at the current bid price, the cluster cannot run jobs.

We have created scripts that create default cluster configuration files for the workloads described in this paper and instructions for copying the appropriate installation files to S3 (see Supplemental Materials).

## Discussion

Because of the cost of scanning subjects and limits on the number of subjects who can be scanned in any given timeframe, neuroimaging workloads at many labs are relatively small. Software has generally been optimized to complete in a reasonable amount of time on commonly used desktops and workstations. Indeed, there may currently be few neuroimaging workloads that currently exceed the capacity provided by a cluster of several workstations, and are thus candidates for the cloud. However, trends toward large-scale data sharing, the availability of higher resolution multiband data and statistical methods that require more computation time might change this. Cloud computing is a viable alternative to on-premises hardware for labs that do not have sustained workloads that would justify the purchase of additional hardware. Even if on-premises computing resources are sufficient to accommodate the workload, the ability to create a cluster on demand enables one to run larger problems in the same amount of time, perhaps on specialized hardware (e.g., GPUs) that are not available in the lab.

Despite the complexity of AWS services, we were able to limit our benchmarking to a small set of candidate applications that would benefit from cloud computing, and a corresponding subset of appropriate instance types. We identified important trends in selection of instance types for lowest cost. First, smaller instance types performed better than larger ones, and c4 instance types performed best (as expected) on the compute intensive workloads. Importantly, we found that we could consistently estimate execution time on EC2 instances from our on-premises workstation. We also outlined how to calculate GPU speedup and to determine whether it is more cost efficient to use a GPU or to parallelize over vCPUs. For our workloads, BEDPOSTX is always faster to run on GPUs versus vCPUS but PROBTRACKX depended on the characteristics of the problem. Dense connectome tractography, which generates thousands of streamlines between locations, achieved higher speedup using the GPU implementation than the thalamic parcellation benchmark, which was smaller and less able to benefit from GPU acceleration. This underscores the importance of benchmarking specific workflows to determine the most cost-effective EC2 platform. Although the nature of neuroimaging applications and cloud services will change in the future, the fundamental approach to benchmarking and making cost calculations described in this paper will remain relevant for understanding any pay as you go computing model.

Finally, we have released some tools developed as a result of our experiences to make the process of estimating the cost of running a workload on EC2 and launching a cluster more streamlined. Because the system administration overhead of setting up machines is automated in EC2, it is possible to think about creating a special purpose cluster of machines optimized for a specific workload. To do this, we leverage AWS cfncluster, which involves creating an install script for specialized software to support a job on an AMI customized for cfncluster. This strategy runs contrary to a common paradigm for creating a lab computing environment, which is to install all necessary software on a computing platform. The need for IT support is therefore minimized because there are fewer interactions between different versions of programs that can cause problems, and troubleshooting is easier. However, in EC2 the most effective strategy is to limit the disk size and avoid any extraneous processes so that resources are dedicated entirely to the neuroimaging workload. A cost-effective strategy is to use Spot pricing and to bid no more than the on-demand pricing (or slightly above), knowing that most hours will incur the Spot price. Note, however, that our cost estimates do not take into account any additional overhead required to create cluster instances and configure cluster software. These contributions reflect the current state of the art and best practices and are likely to change significantly as AWS services evolve.

In summary, we believe that cloud computing will play an increasingly important role in neuroimaging. It is easy to conjecture that neuroimaging applications will change rapidly, as will cloud services, and that in the future they may not resemble those described in this paper. However, as long as there is a cost associated with application execution time, the benchmarking approach described here can be used to guide decisions about use and selection of cloud services and investment in on-premises resources. In the meantime, the strategies and software we have outlined contribute to current best practices for use of AWS computing services.

## Author Contributions

TM and TG conceived of and designed this project and analysis. TM, NK, and TKMD wrote supporting software and documentation. All authors contributed significantly to the acquisition and analysis of data, drafting and revising the manuscript, and have given final approval and are accountable for the accuracy and integrity of this work.

## Conflict of Interest Statement

The authors received credits from Amazon Web Services Cloud Credits for Research program to support this research project. Amazon Web Services had no role in the experimental design, data collection, analysis, or writing of this research.

## Footnotes

1 http://cfncluster.readthedocs.io/en/latest/. Note that cfncluster is the successor to starcluster (http://star.mit.edu/cluster/)

2 https://github.com/IBIC/ibic-cfncluster

3 https://aws.amazon.com/ec2/instance-types

4 http://adni.loni.usc.edu/

5 http://fcon_1000.projects.nitrc.org/

6 http://imaging.ukbiobank.ac.uk/

7 http://www.humanconnectomeproject.org/

8 https://aws.amazon.com/ec2/instance-types/

9 https://adni.loni.usc.edu

10 http://depts.washington.edu/adrcweb/

11 https://aws.amazon.com/ec2/instance-types/

12 Note that to assemble the input data for this problem size, it was necessary to use a large G4 instance with sufficient memory. However, our speedup calculations do not take into account memory as a limiting factor.

13 https://arc.liv.ac.uk/trac/SGE
