# Supplementary material for: Running Neuroimaging Applications on Amazon Web Services: How, When, and at What Cost?
Source: Front Neuroinform. 2017 Nov 3;11:63. doi: 10.3389/fninf.2017.00063 (PMC5675877; doi:10.3389/fninf.2017.00063)

Specifications for cost models in Figure 1

Desktop:

We approximate $1000 for a quad core Dell Intel (R) Core™ i7 processor and at least 8GB memory and 1TB disk.

Workstation:

The workstation that we benchmarked was obtained from Silicon Mechanics. This workstation is a GPU-capable 2U server, outfitted with

- CPU: 2 Intel® Xeon® processors E5-2680 v3, with 24 total cores at 2.5 GHz
- Memory: 256GB RAM (16 x 16GB DDR4-2133 RDIMMs), operating at 1866 MT/s
- Interconnect: 10 gigabit Ethernet (onboard dual-port Intel® X540 10GBase-T)
- Storage: 60TB SAS3 drives, 7.2K RPM, configured as RAID 10 with Hot Spare using SAS3 HBA in IT mode
- Boot Drive: 1 Intel® 240GB DC S3510 MLC SATA SSD
- OS: latest version of Debian

Cost: Purchased 12/2015 for $12,807. We assume a service contract of approximately $250/year and 5% system administrator paid at $10,700/month. This salary was estimated by extrapolating hourly UW-IT rates.

Configuration notes:

We chose to use the processor generation that was one behind the latest to maximize price/performance while remaining up to date.

The workstation is outfitted with sufficient memory to handle most workloads that we run in our lab. It would be possible to increase the memory at a significant cost to accommodate memory intensive applications.

We did not outfit this workstation with GPUs but this form factor is capable of accommodating two GPUs.

We chose to use RAID 10 to configure our drives, trading raw capacity and availability in the event of two simultaneous disk failures for array rebuild speed. The operating system is stored on an SSD drive to improve speed, particularly for neuroimaging applications that make heavy use of temporary files.

We selected a 2U unit to minimize cost and space to mount this workstation in a shared server rack. However, similar configurations can be made in a tower form factor, which does not require a rack.

We specifically run Debian Linux to benefit from the Neurodebian repository, reducing system administrator overhead required to manage our machines. We have found that compared to Ubuntu, Debian is lighter weight and more stable. We use the XFCE window system to maximize stability and speed over remote X connections.

Cluster:

Cost estimates are based on the University of Washington Scalable Cluster solution (Hyak): https://itconnect.uw.edu/service/shared-scalable-compute-cluster-for-research-hyak/

Briefly, Hyak offers CPU and storage options for high performance computing. All Hyak use is mediated by a system scheduler, TORQUE, that allows users to run interactive, batch and parallel jobs. Compute nodes, each consisting of 28 cores, operate for four years when purchased. Hyak currently comprises 1100 cores with 250 TF performance, but is the midst of upgrading to a new cluster system with 9000 cores and 300 TF performance.

Assuming a four year Hyak reservation, effectively hourly rates, intended to approximate AWS c3.8xlarge performance, are as follows (Personal communication with Robert Fatland, UW Director of Cloud Computing):

% Utilization	Hyak hourly rate
100%	$0.14
90%	$0.15
80%	$0.17
70%	$0.20
60%	$0.23
50%	$0.28
40%	$0.35
30%	$0.46
20%	$0.69
10%	$1.38

Equations for neuropointillist

The two models tested and compared using the `anova` function in R were:

```
mod1 <- lme(Y ~ age+ time + domain + target, random=~1+time|idnum, method=c("ML"),
na.action=na.omit, control=lmeControl(returnObject=TRUE,sing
ular.ok=TRUE)))

mod2 <- lme(Y ~ age + time + domain + target + target*domain, random=~1+time|idnum,
method=c("ML"), na.action=na.omit, control=lmeControl(returnOb
ject=TRUE,singular.ok=TRUE)))
```

Configuring CfnCluster

Below is a multi-step process that describes everything you have to do to create an AWS cluster using CfnCluster. Although it is daunting, you only need to go through this procedure once. The goal is to create a working configuration file that you can modify with your own instance type and spot price.

1. Create an AWS account if you do not have one, and obtain your access key ID and secret access key. Follow directions on Sign Up for Amazon Web Services here:

http://docs.aws.amazon.com/AWSEC2/latest/UserGuide/get-set-up-for-amazon-ec2.html#sign-up-for-aws

When you select Download Credentials, note where you store these keys. You will need this to create a cfncluster.

2. Install the AWS command line interface (CLI), if it is not already installed on your computer system.

You will probably find it easier to interact with AWS from the command line. The AWS CLI is also a prerequisite for these scripts.

Follow directions on Installing the AWS Command Line Interface to install the AWS CLI.
http://docs.aws.amazon.com/cli/latest/userguide/installing.html

3. Configure the AWS CLI for use.

The AWS CLI should be configured for use with your AWS access key ID and secret access key, downloaded to a secure location in step 1.

You should also select a default region. In the AWS console, you will choose a region to the right of your name (**Figure 1**). You will need to convert this region name to a code. The lookup table for region names to codes may be found here:

http://docs.aws.amazon.com/AWSEC2/latest/UserGuide/using-regions-availability-zones.html#concepts-available-regions

In this example we will use Oregon, or us-west-2 as the default region.

Finally, you can select a default output format. Useful output formats are json or text. In this example we use text as the default output format.

Use the configure command as below to configure your AWS CLI.

```
# aws configure
AWS Access Key ID [None]: INSERTYOURACCESSKEYHERE
AWS Secret Access Key [None]: INSERTYOURSECRETACCESSKEYHERE
Default region name [None]: us-west-2
Default output format [None]: text
```

You may reconfigure the AWS CLI later, or override settings for region or output format using command flags.

4. Sign up for Amazon S3 if you have not, and create a bucket for storage of data.

Amazon's instructions are available here (follow the first two steps).
http://docs.aws.amazon.com/AmazonS3/latest/gsg/GetStartedWithS3.html

For the purposes of this tutorial we will assume you have created a bucket called <user>-s3 in the default region that you specified in step 3, where <user> is your user name. In this example I will refer to tara-s3, which is my bucket. You will need to change this to be your bucket; note that in commands it is highlighted in red to remind you to edit it. The namespace for buckets is shared across all users, so your bucket name must be unique. Make a note of your bucket name to modify commands below.

5. Create an AWS key pair

This AWS key pair is different from the AWS access key and AWS secrete access key in (3). To securely create a cluster with your credentials that only you can log in to, you will first need to create an AWS key pair. You can do this following the instructions below. As described, save your private key (with the .pem extension) to a safe place and make sure that only you can read it. If you do not do this, you will have difficulty logging on to your cluster!

http://docs.aws.amazon.com/AWSEC2/latest/UserGuide/ec2-key-pairs.html - having-ec2-create-your-key-pair

6. Configure CfnCluster

With your key from step 3 nearby, configure CfnCluster:

```
cfncluster configure
```

You will need to provide the following:
AWS Access Key ID (as in step 3)
AWS Secret Access Key ID (as in step 3)
AWS Region ID (as in step 3)
VPC Name (Use the default here, "public")
Key Name (the name you used in step 5)
VPC ID (use one of the acceptable values here)

Master Subnet ID (use one of the acceptable values here)

This will create a file called ~/.cfncluster/config. Copy this file to ~/.cfncluster/config.orig; you will need to grab the VPC settings from it later.

7. Upload configuration scripts and the resources that they need to your S3 bucket.

Note: Storage in S3 incurs a monthly charge. As of writing, this currently approximates $0.03 per gigabyte (https://aws.amazon.com/s3/pricing/). Do not upload large files to S3 to support FSL or FreeSurfer as described below if you do not intend to use them on your cluster.

FSL Specific Instructions

1. Follow the directions under Advanced Usage, Manual installation to download a tar.gz file of FSL for Centos.
http://fsl.fmrib.ox.ac.uk/fsl/fslwiki/FslInstallation/Linux

You do not need to unpack this distribution.

2. Copy this tar.gz file to your tara-s3 bucket:

```
aws s3 cp fsl-5.0.9-centos6_64.tar s3://tara-s3/fsl-5.0.9-
centos6_64.tar
```

FreeSurfer Specific Instructions

1. Download the FreeSurfer distribution for Centos (http://freesurfer.net/fswiki/Download)

2. Copy this tar.gz file to your tara-s3 bucket:

```
     aws s3 cp freesurfer-Linux-centos6_x86_64-stable-pub-
v5.3.0.tar.gz s3://tara-s3/freesurfer-Linux-centos6_x86_64-
stable-pub-v5.3.0.tar.gz
```

3. You will need a license key to operate FreeSurfer. Save your license key in a file called license.txt and copy it to your tara-s3 bucket.

```
aws s3 cp license.txt s3://tara-s3/license.txt
```

8. Install ibic-cfncluster

The software to estimate cost and to set up and configure a cfncluster as decribed in this paper is in the github repository

https://github.com/IBIC/ibic-cfncluster.git

Clone this repository as follows:

git clone https://github.com/IBIC/ibic-cfncluster.git

The programs that are used to estimate cost or to create a cfncluster configuration file will be located in

```
ibic-cfncluster/bin
```

There are two versions of the cost estimator, one written in R and one written in Python. To use the Python cost estimator, you will need to install boto 3, the AWS SDK for Python. See

https://boto3.readthedocs.io/en/latest/

for instructions to install boto 3. To use the R version of the cost estimator, you will need to have R and the argparse library installed.

https://cran.r-project.org/web/packages/argparse/index.html

See the README in the github repository for the latest instructions for running these programs.

9. Configure the FSL and FreeSurfer installation scripts and copy them to S3

If you have copied FSL or FreeSurfer executables to S3, you will need to configure shell scripts to install these packages on the cfncluster when it starts. You can do this by running the following command, replacing tara-s3 with your own bucket name.

```
ibic-cfncluster/bin/install_s3_scripts tara-s3
```

10. Identify the most cost-effective instance type for your cluster.

We assume that you have identified some reference machine (e.g., the workstation referred to in the paper) and have benchmarked your application both on this reference machine and on relevant EC2 instance types, so that you can estimate the ratio of execution time on the reference machine to vCPU hours (as shown in Table 1). You will need to estimate the total number of vCPU hours required by each job, and the number of jobs that you wish to run.

Using these inputs, identify the most cost-effective instance type using ibic-get-spot-estimate.

For example, if you estimate that each job will take 36 hours, and there are 500 jobs, you would run (the syntax is the same for both versions):

```
ibic-get-spot-estimate --hours 36 --num 500
```

You will obtain a cost estimate for running the job on an EC2 cluster (based on the average Spot price for the preceding week) and the least expensive instance type. Note that you need to ensure that your application will run on the suggested instance type. If additional storage or memory are required to run your application these will incur an additional cost that is not factored in to this estimate. Also, there is no guarantee that Spot pricing will not increase radically from week to week. Finally, note that in our experience, it has taken a variable amount of time to launch cfncluster. If this is an issue for you, you may want to consider factoring in an additional overhead cost.

11. Create a custom cluster to run your application.

Finally, you can create a cluster to run your application. The first step to doing this is to create a configuration file for cfncluster in which you specify the instance type identified in (7) and the name of the key pair that you created in (8). You only need to do this once- it will be obvious where to change the name of the instance type and spot price.

```
ibic-create-cluster-config --instancetype <type> --keypair <privatekey>
```

By default, this will create a configuration file in `~/.cfncluster/config` with configuration templates for clusters to run freesurfer, fsl, and R (default).

However, you need to add to this configuration file the information about your VPC that you saved in ~/.cfncluster/config.orig in (6). Copy the three lines from that file of the form:

```
[vpc public]
master_subnet_id = subnet-xxxxxxxx
vpc_id = vpc-xxxxxxxxx
```

into the configuration file that you just generated.

12. Start your selected cluster.

To start the default cluster (using the name mycluster; you can provide any name)

```
cfncluster create mycluster
```

Alternatively, to start a cluster pre-loaded with software for an FSL analysis:

```
cfncluster --cluster-template fsl create mycluster
```

13. Run your analysis

After creating the cluster as above, the cfncluster startup procedure will print out a public IP address. To connect to this cluster, you will need to specify the file name containing your private key. Once you have logged in, you can run your analyses as you would with any cluster!

```
ssh -i <privatekey> ec2-user@IP-ADDRESS-OF_CLUSTER
```

14. Terminate your cluster

Once you have finished executing your job and collected your results, you should terminate your cluster to avoid being charged for time you are not using. Log out and return to the shell from which you started your cluster (12).

```
cfncluster delete mycluster
```

Figure 1.
The EC2 dashboard showing at top right, circled, the region name (Oregon). Instances and storage is generally located in a region that includes one or more data centers (called Availability Zones).

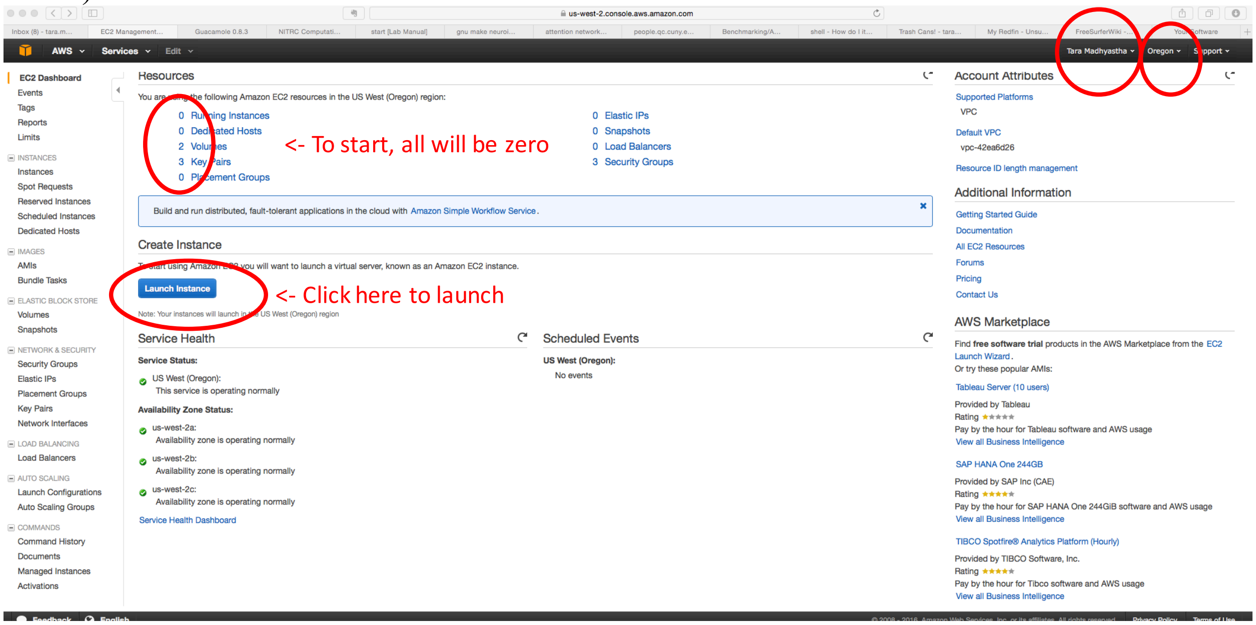

Supplement: Supplementary file 1 [file Presentation_1.PDF]
